# Mediterranean diet and quality of life in women treated for breast cancer: A baseline analysis of DEDiCa multicentre trial

**DOI:** 10.1371/journal.pone.0239803

**Published:** 2020-10-08

**Authors:** Giuseppe Porciello, Concetta Montagnese, Anna Crispo, Maria Grimaldi, Massimo Libra, Sara Vitale, Elvira Palumbo, Rosa Pica, Ilaria Calabrese, Serena Cubisino, Luca Falzone, Luigina Poletto, Valentina Martinuzzo, Melania Prete, Nadia Esindi, Guglielmo Thomas, Daniela Cianniello, Monica Pinto, Michelino De Laurentiis, Carmen Pacilio, Massimo Rinaldo, Massimiliano D’Aiuto, Diego Serraino, Samuele Massarut, Chiara Evangelista, Agostino Steffan, Francesca Catalano, Giuseppe L. Banna, Giuseppa Scandurra, Francesco Ferraù, Rosalba Rossello, Giovanna Antonelli, Gennaro Guerra, Amalia Farina, Francesco Messina, Gabriele Riccardi, Davide Gatti, David J. A. Jenkins, Anita Minopoli, Bruna Grilli, Ernesta Cavalcanti, Egidio Celentano, Gerardo Botti, Maurizio Montella, Livia S. A. Augustin

**Affiliations:** 1 Epidemiology and Biostatistics Unit, Istituto Nazionale Tumori—IRCCS "Fondazione G. Pascale", Napoli, Italy; 2 Department of Biomedical and Biotechnological Sciences Oncologic, Clinical and General Pathology Section, University of Catania, Catania, Italy; 3 Department of Clinical Medicine and Surgery, Federico II University, Napoli, Italy; 4 Cancer Epidemiology Unit, National Cancer Institute Centro di Riferimento Oncologico, IRCCS, Aviano, Italy; 5 School of Life and Environmental Sciences and Charles Perkins Centre, University of Sydney, Sydney, Australia; 6 Clinica Mediterranea, Napoli, Italy; 7 Division of Breast Oncology, Istituto Nazionale Tumori—IRCCS "Fondazione G. Pascale", Napoli, Italy; 8 Rehabilitation Medicine Unit, Istituto Nazionale Tumori—IRCCS "Fondazione G. Pascale", Napoli, Italy; 9 Clinica Villa Fiorita, Aversa, Italy; 10 Division of Breast Cancer Surgery, Centro di Riferimento Oncologico, IRCCS, Aviano, Italy; 11 Immunopathology and Cancer Biomarkers Unit, National Cancer Institute Centro di Riferimento Oncologico IRCCS, Aviano, Italy; 12 Cannizzaro Hospital, Catania, Italy; 13 Ospedale San Vincenzo, Taormina, Italy; 14 Ospedale Evangelico Betania, Napoli, Italy; 15 Rheumatology Unit, University of Verona, Verona, Italy; 16 Department of Nutritional Sciences, Faculty of Medicine, University of Toronto, Toronto, Ontario, ON, Canada; 17 Clinical Nutrition and Risk Factor Modification Centre, St. Michael's Hospital, Toronto, Ontario, ON, Canada; 18 Li Ka Shing Knowledge Institute, St. Michael's Hospital, Toronto, Ontario, ON, Canada; 19 Laboratory Medicine Unit, Istituto Nazionale Tumori—IRCCS "Fondazione G. Pascale", Napoli, Italy; 20 Scientific Directorate, Istituto Nazionale Tumori—IRCCS "Fondazione G. Pascale", Napoli, Italy; University of Zurich, SWITZERLAND

## Abstract

Evidence suggests a beneficial role of the Mediterranean Diet (MedDiet) on health-related quality of life (HRQoL) in healthy subjects. HRQoL is relevant in cancer therapy and disease outcomes, therefore we investigated the association between adherence to the MedDiet and HRQoL in breast cancer survivors participating in the multicentre trial DEDiCa. Diet and HRQoL were assessed at baseline in a subgroup of 309 women enrolled within 12 months of breast cancer diagnosis without metastasis (stages I-III, mean age 52±1 yrs, BMI 27±7 kg/m^2^). The 14-item PREDIMED questionnaire was used to analyse adherence to the MedDiet. HRQoL was assessed with three validated questionnaires measuring physical, mental, emotional and social factors: EQ-5D-3L, EORTC QLQ-C30 and EORTC QLQ-BR23. Analysis of variance (ANOVA) and multivariate analyses were performed to assess the possible role of the MedDiet on HRQoL. Patients with higher adherence to MedDiet (PREDIMED score >7) showed significantly higher scores for physical functioning (p = 0.02) and lower scores on the symptomatic pain scale (p = 0.04) assessed by the EORTC QLQ-C30 questionnaire compared to patients with a lower adherence to MedDiet (PREDIMED score ≤7). Higher scores from the EQ-5D-3L indicating higher well-being were observed mainly in participants with higher MedDiet adherence (p = 0.05). In adjusted multivariate analyses significant positive associations were found between MedDiet, physical functioning (p = 0.001) and EQ 5D-3L score (p = 0.003) while inverse associations were found with pain and insomnia symptoms (p = 0.005 and p = 0.029, respectively). These results suggest that higher adherence to the MedDiet in breast cancer survivors is associated with better aspects of quality of life, specifically higher physical functioning, better sleep, lower pain and generally higher well-being confirming findings in healthy subjects.

## Introduction

Several lines of evidence indicate the beneficial role of the Mediterranean Diet (MedDiet) on total mortality and on primary and secondary prevention of chronic disease, such as diabetes, cardiovascular disease and cancer including breast cancer [1–4]. Breast cancer is the most common cancer among women worldwide [5]. Poor diet and lack of physical activity are risk factors for breast cancer development and mortality while lifestyle interventions with healthy diets and increased physical activity may be of benefit [6–8]. Higher adherence to the MedDiet has been associated with a reduced risk of breast cancer [1, 9–14] and better health-related quality of life (HRQoL) in healthy subjects [15, 16]. HRQoL generally is an indication of the impact of a medical condition or treatment on a person’s physical condition, emotional state and social factors [17].

Self-perceived HRQoL may be a predictor of mortality [18, 19] and could be influenced by dietary patterns [16]. Cancer patients report declines in physical functioning, increased pain and generally reduced quality of life [20–22] which could reduce compliance to oncologic treatment with potential consequences on cancer prognosis and mortality [23, 24]. No studies investigated the association between adherence to the MedDiet and HRQoL in patients diagnosed with breast cancer albeit evidence suggests that high consumption of key components of the MedDiet such as vegetables and fruit, were positively associated with HRQoL in breast cancer survivors. In particular breast cancer survivors who ate more than 250 g/day of vegetables and fruit showed better quality of life than those eating less than 250 g/day [25].

We investigated the possible role of the MedDiet in HRQoL in breast cancer survivors participating in a dietary intervention trial in Italy.

## Materials and methods

### Design

This study is part of an ongoing multicenter randomized controlled trial of the effectiveness of a treatment program including dietary modification, physical activity and vitamin D supplementation (DEDiCa Study) on breast cancer recurrence [26]. The study protocol was approved by the Italian Ministry of Health, Italian Medicine Agency (AIFA) and the Ethic Boards of each recruiting hospital (ClinicalTrials.gov NCT02786875). Participants were recruited and followed up in national cancer institutes or oncologic departments of hospitals located in Southern and Northern Italy: Istituto Nazionale Tumori IRCCS Fondazione G. Pascale (Naples), Clinica Mediterranea (Naples), Villa Betania (Naples), Cannizzaro Hospital (Catania), San Vincenzo Hospital (Taormina), Istituto Nazionale Tumori IRCCS CRO (Aviano). Eligible participants were found through surgical lists of participating hospitals. They were contacted by phone and offered to learn more about the study during group information sessions. Informed consent was obtained at baseline from all participants included in the study.

A total of 309 women with breast cancer were recruited in the study from November 2016 to April 2019, based on the following inclusion criteria: women with primary diagnosis of histologically confirmed breast cancer (T1 with Ki67 ≥ 30%, T2, T3 without metastasis) within 12 months from diagnosis; age ≥30 and <75 years; patients who are able to comprehend and are willing to sign the consent form and are able to adhere to the protocol including scheduled clinic visits and assigned treatment. The exclusion criteria were: patients who do not possess the inclusion criteria for this study; patients with sarcoidosis or other granulomatous diseases or with hypercalcemia (Ca >11 mg/dL); patients with any previous or current concomitant malignant cancer; pregnant or lactating women; patients with AIDS diagnosis; patients with severe renal insufficiency; patients with kidney stones (nephrocalcinosis or nephrolithiasis); patients participating in other lifestyle clinical trials) [26].

Demographic characteristics of the 309 participants are shown in Table 1.

**Table 1:** Demographic characteristics of the participants (n = 309).

	n (%)
**Age**	
*52*.*0 ± 9*.*2* (*mean±SD)*	
<40 yrs	29 (9.4)
41–50 yrs	125 (40.5)
51–60 yrs	93 (30.1)
>60 yrs	62 (20.1)
**Civil Status**	
*Married (or common law)*	248 (80.8)
*Single*	59 (19.2)
**Education**	
*≤ 11 yrs*	111 (36.0)
*≥ 12 yrs*	197 (64.0)
**Tumor size (T)**	
*T1*	195 (63.1)
*T2/T3*	114 (36.9)
**Lymph node status (N)**	
*N0*	145 (46.9)
*N1*	121 (39.2)
*N2*	35 (11.3)
*N3*	8 (2.6)
**Cancer stage**	
*I*	93 (30.1)
*IIA*	132 (42.7)
*IIB*	40 (12.9)
*IIIA*	36 (11.7)
*IIIC*	8 (2.6)
**Body mass index (BMI)**^a^	
*27*.*6 ± 6*.*0* (*mean±SD)*	
Normal weight	127 (41.1)
Overweight	88 (28.5)
Obese	94 (30.4)
**PREDIMED Score**^b^	
*7*.*9 ± 1*.*9* (*mean±SD)*	
Low adherence MedDiet (*≤*7)	137 (44.3)
High adherence MedDiet (>7)	172 (55.7)

^a^Normal weight <25.0 kg/m^2^, Overweight 25.0–29.9 kg/m^2^, Obese ≥30.0 kg/m^2^;

^b^Single are widow, divorced or maiden. Predimed 14-item score questionnaire: score ≤7 low adherence to Mediterranean Diet; score >7 high adherence to Mediterranean Diet

Questionnaires were administered at baseline to assess sociodemographic factors, adherence to the MedDiet and quality of life (details of these questionnaires are explained below). Weight and height of study participants were obtained by trained staff at the baseline study visits. Height was measured to the nearest 1 cm using a Seca stadiometer and weight was measured to the nearest 0.5 Kg using a Seca scale (Seca 761). Body mass index (BMI) was calculated using the formula weight (kg) / height (m^2^). Physical activity level was evaluated with a step counter (Omron Walking Style IV) provided by study staff prior to the baseline visits.

### Instruments

#### Adherence to mediterranean diet

At baseline all participants completed the 14-item PREDIMED questionnaire administered by the study staff. This questionnaire has been used in other studies assessing adherence to the MedDiet in people living in the Mediterranean basin [27].

The PREDIMED questionnaire consists of 14 questions: 12 questions on food quantities and frequency of consumption (olive oil, vegetables, fruit, red or processed meats, butter, soda drinks, legumes, fish, commercial sweets, nuts, wine, sofrito sauce) and 2 general questions on food intake habits on olive oil and meat. Each question included two possible answers and scores; 1 score for “yes” and 0 for “no”.

The PREDIMED final score can ranges from 0 to 14 where 14 represented the highest adherence to MedDiet.

#### Health Related Quality of Life (HRQoL)

Three self-assessed questionnaires were completed: the European Quality of Life 5 Dimensions 3 Level (EQ-5D-3L) [28], the European Organization for Research and Treatment of Cancer Quality of Life Questionnaire Core 30 items (EORTC QLQ-C30) and Breast Cancer 23 items (EORTC QLQ-BR23) [29].

Questionnaire EQ-5D-3L is a non-cancer-specific measure of generic health status that includes a descriptive system comprising five dimensions (mobility, self-care, usual activities, pain or discomfort, and anxiety or depression) and three levels of perceived problems (1 for no problems, 2 for some problems and 3 for extreme problems). A unique health index score is calculated by applying an algorithm that attaches coefficients (called weights) to each value of the levels for each dimension; we have chosen the Italian Model to estimate EQ-5D-3L index score [30]. In addition, a visual analogue scale (EQ VAS) included in this questionnaire measures self-perceived health status as a numeric percentage from 0 (worst health status) to 100 (best health status).

The EORTC QLQ-C30 (Questionnaire for Quality of Life Assessment in patients with cancer, version 3.0) measures five functional dimensions (physical, role, emotional, cognitive and social), three symptom items (fatigue, nausea or vomiting, and pain), six single items (dyspnoea, sleep disturbance, appetite loss, constipation, diarrhoea, and financial impact), and a global health status/QoL which is the mean of two questions regarding overall health and overall quality of life.

The questionnaire consists of 30 items and can be used in all patients receiving cancer treatment regardless of cancer type and location. The EORTC QLQ-BR23 (Quality of Life Questionnaire—Breast Cancer) is a breast cancer-specific module that comprises 23 questions to assess body image, sexual functioning, sexual enjoyment, future perspective, systemic therapy side effects, breast symptoms, arm symptoms and distress from hair loss.

Scoring of the EORTC QLQ-C30 and QLQ-BR23 were performed according to the EORTC QLQ-C30 Scoring Manual 3^rd^ Edition. All scores from the individual parts of the questionnaire range from 1 to 4. All scores were linearly transformed to a 0–100 scale. Higher scores for functioning and for global health status indicate better health. Conversely, higher scores for symptoms indicate worse health.

In our study we did not include analyses related to sexual enjoyment and feeling towards hair loss because 62% and 77% of the participants, respectively, did not respond to this scale.

#### Statistical analysis

Baseline characteristics were described as number (n) and percentage (%). In each HRQoL dimension we compared the means of the two categories of adherence to the MedDiet, high (score >7) and low, with univariate analysis of variance (ANOVA). Effect size was evaluated using standardized means difference method. In order to take into consideration multiple comparisons, multiple linear regression adjusted models were performed to assess the association between baseline MedDiet adherence and HRQoL dimensions. Covariates included in the basic model were age (continuous) and cancer stage (I, II and III); this model was additionally adjusted for BMI (continuous), type of surgery (quadrantectomy, mastectomy), comorbidities (0, 1, ≥2) and combined therapy (none, at least one) in the multivariate adjusted model I. A multivariate adjusted model II was also considered which included the basic model with further adjustments for smoking status (no, yes, former), step count (continuous), education (continuous) and civil status (married or single). Results are reported as beta regression coefficients.

The Statistical Package for the Social Sciences (SPSS) software, version 25.0 (Chicago, IL, USA) was used for all data analyses. Results were considered statistically significant at a p-value <0.05.

## Results

Baseline characteristics of randomized participants (n = 309) are shown in Table 1. Mean age (±SD) was 52.1 (±9.2) years: 54% were ≥50 years and 46% were <50 years. Mean body mass index (BMI) was 27.7 kg/m^2^ (±6.0), of which 28.5% were overweight (BMI between 25 and 29.9) and 30.4% were obese (BMI greater than or equal to 30). The majority of participants were married or common law (80.8%) and 64% had attained a high school or higher education. Additional descriptive data for socio-demographic and cancer related characteristics are shown in Table 2.

**Table 2:** Baseline characteristics of the participants (n = 309) according to baseline categories of adherence to Mediterranean Diet (MedDiet) assessed by PREDIMED 14-item questionnaire.

	Low Adherence MedDiet (≤7) n = 137		High Adherence MedDiet (>7) n = 172		
	*n*	*(%)*	*n*	*(%)*	*p value*
**Age (years)**					
*<50 yrs*	67	(48.9)	75	(43.6)	*0*.*35*
*≥50 yrs*	70	(51.1)	97	(56.4)	
**Body Mass Index (kg/m**^**2**^**)**^a^					
*Normal weight*	52	(38.0)	75	(43.6)	*0*.*57*
*Overweight*	40	(29.2)	48	(27.9)	
*Obese*	45	(32.8)	49	(28.5)	
**Physical activity (steps/day)**^b^					
*Sedentary*	66	(46.8)	75	(53.2)	*0*.*46*
*Low active*	41	(43.6)	53	(56.4)	
*Active or highly active*	26	(19.5)	43	(25.1)	
**Smoking status**					
*No smoker*	59	(44.0)	93	(54.4)	*0*.*09*
*Smoker*	32	(23.9)	26	(15.2)	
*Former smoker*	43	(32.1)	52	(30.4)	
**Civil status**					
*Married (or common law)*	117	(86.7)	131	(76.2)	***0*.*02***
*Single*^c^	18	(13.3)	41	(23.8)	
**Education (years of school)**					
*≤ 11 yrs*	62	(45.6)	49	(28.5)	***0*.*02***
*≥ 12 yrs*	74	(54.4)	123	(71.5)	
**Number of comorbidities**^d^					
*0*	87	(63.5)	100	(83.1)	*0*.*52*
*1*	36	(26.3)	48	(27.9)	
*≥2*	14	(10.2)	24	(14.0)	
**Type of surgery**					
*Quadrantectomy*	101	(74.3)	134	(78.4)	*0*.*40*
*Mastectomy*	35	(25.7)	37	(21.6)	
**Time from surgery**					
*< 8 months*	87	(63.5)	93	(54.1)	*0*.*09*
*> 8 months*	50	(36.5)	79	(45.9)	
**Cancer treatment**					
*Adjuvant chemotherapy*					
*Current*	21	(15.6)	29	(17.6)	*0*.*42*
*Not current*	60	(44.4)	82	(49.7)	
*Never*	54	(40.0)	54	(32.7)	
*Radiotherapy*					
*Current*	12	(9.2)	11	(6.7)	*0*.*60*
*Not current*	60	(46.2)	83	(50.9)	
*Never*	58	(44.6)	69	(42.3)	
*Hormone therapy*					
*Current*	72	(55.8)	90	(55.9)	*0*.*97*
*Not current*	2	(1.6)	2	(1.2)	
*Never*	55	(42.6)	69	(42.9)	
*Biological therapy*					
*Current*	20	(14.8)	26	(15.5)	*0*.*53*
*Not current*	1	(0.7)	0	(0.0)	
*Never*	114	(84.4)	142	(84.5)	

^a^Normal weight <25.0 kg/m^2^, Overweight 25.0–29.9 kg/m^2^, Obese ≥30.0 kg/m^2^;

^b^Physical activity (steps/day): Sedentary <5000, Low active 5000–7500, Active or highly active>7500;

^c^Single are widow, divorced or maiden;

^d^Type 2 diabetes, hypertension, hypertriglyceridemia, hypercholesterolemia. n = number.

Adherence to the MedDiet was categorized as low (score ≤7) and high (score >7) adherence. Married and more educated patients had a higher adherence to MedDiet (p = 0.02), as shown in Table 2. Table 3 shows the mean scores of the different dimensions of HRQoL questionnaires according to categories of baseline adherence to the MedDiet.

**Table 3:** Mean scores (SD) of HRQoL questionnaires (EORTC QLQ-C30, EORTC QLQ-BR23, EQ-5D-3L) according to categories of adherence to Mediterranean Diet in breast cancer survivors.

	Low Adherence MedDiet (≤7) n = 137		High Adherence MedDiet (>7) n = 172			
	*n*	*mean±SD*	*n*	*mean±SD*	*Effect size*	p value*
**EORTC QLQ-C30**						
***Functioning***						
*Physical functioning*	137	78.9±17.8	171	83.3±14.5	0.28	***0*.*02***
*Role functioning*	137	78.5±24.3	172	80.0±22.8	0.07	*0*.*56*
*Emotional functioning*	137	71.8±21.2	172	75.3±251.6	0.01	*0*.*15*
*Cognitive functioning*	137	81.4±21.7	172	80.8±21.5	0.02	*0*.*82*
*Social functioning*	137	75.7±25.7	172	76.9±25.9	0.04	*0*.*67*
***Symptoms***						
*Fatigue*	137	35.0±23.5	172	32.9±23.5	0.08	*0*.*42*
*Nausea and vomiting*	137	6.9±14.2	172	7.8±13.3	0.06	*0*.*60*
*Pain*	137	28.5±24.3	172	23.1±21.7	0.23	***0*.*04***
*Dyspnea*	136	21.6±23.1	171	18.12±22.9	0.15	*0*.*19*
*Insomnia*	135	32.8±27.6	172	26.7±28.3	0.21	*0*.*06*
*Appetite loss*	136	7.6±15.1	172	6.4±17.4	0.07	*0*.*52*
*Constipation*	128	14.3±23.5	149	15.0±21.4	0.03	*0*.*81*
*Diarrhoea*	136	10.0±17.4	171	7.4±15.7	0.15	*0*.*16*
*Financial*	136	19.1±27.1	170	19.0±27.3	0.03	*0*.*97*
*Global Health Status/QoL*	135	63.2±20.6	172	62.9±22.1	0.01	*0*.*93*
**EORTC QLQ-BR23**						
***Functioning***						
*Body image*	136	60.6±29.9	172	65.6±30.6	0.16	*0*.*15*
*Sexual functioning*	135	81.5±22.4	170	80.8±22.1	0.03	*0*.*78*
*Future perspective*	135	42.0±34.1	172	45.6±33.6	0.10	*0*.*36*
***Symptoms***						
*Systematic therapy side effects*	137	26.2±18.9	172	23.9±18.1	0.12	*0*.*29*
*Breast symptoms*	135	24.1±20.0	172	20.2±18.1	0.20	*0*.*08*
*Arm symptoms*	136	21.7±20.4	171	21.1±19.0	0.03	*0*.*79*
**EQ-5D-3L**						
EQ-5D-3L Score	137	0.84±0.12	171	0.87±0.11	0.22	*0*.*05*

*by ANOVA (significance p<0.05), SD, standard deviation; EORTC QLQ-C30, European Organization for Research and Treatment of Cancer Quality of Life Questionnaire Core 30 items and EORTC QLQ-BR23, Breast Cancer 23 items, EQ-5D-3L, European Quality of Life 5 Dimensions-3 Level, MedDiet, Mediterranean Diet.

In the EORTC QLQ-C30 questionnaire we observed that patients with higher adherence to the MedDiet had significantly higher scores for physical functioning (83.3; p = 0.02) and reduced pain symptom (23.1; p = 0.04) scores compared to participants in the lower adherence group (respectively 78.9 and 28.5). Furthermore, we observed that patients with higher adherence to MedDiet showed higher scores of overall wellbeing in the EQ-5D-3L questionnaire compared to participant in the lower adherence group (0.87 vs 0.84; p = 0.05).

The effect size was between small and medium (i.e. >0.20 <0.50) for physical functioning, pain and well-being (0.28, -0.23 and 0.22, respectively) (Table 3).

Similar results were observed in the multivariate analysis (Tables 4 and 5).

**Table 4:** Association between adherence to the Mediterranean Diet (MedDiet) and HRQoL questionnaires EORTC QLQ-C30.

Questionnaire	Adherence to MedDiet assessed by PREDIMED Questionnaire			
**EORTC QLQ C30**				
**Functioning**		*beta*	*p value*	*R*^*2*^
*Physical functioning*	*Age and Cancer Stage Adjusted*	0.199	***0*.*001***	*0*.*038*
	Multivariable Adjusted I*	0.207	***0*.*001***	*0*.*068*
	Multivariable Adjusted II**	0.169	***0*.*006***	*0*.*059*
*Role functioning*	*Age and Cancer Stage Adjusted*	0.060	*0*.*296*	*0*.*004*
	Multivariable Adjusted I*	0.052	*0*.*382*	*0*.*033*
	Multivariable Adjusted II**	0.037	*0*.*534*	*0*.*036*
*Emotional functioning*	*Age and Cancer Stage Adjusted*	0.067	*0*.*247*	*0*.*004*
	Multivariable Adjusted I*	0.059	*0*.*973*	*0*.*033*
	Multivariable Adjusted II**	0.033	*0*.*587*	*0*.*036*
*Cognitive functioning*	*Age and Cancer Stage Adjusted*	0,036	*0*.*535*	*0*.*001*
	Multivariable Adjusted I*	0.036	*0*.*549*	*0*.*031*
	Multivariable Adjusted II**	0.011	*0*.*854*	*0*.*035*
*Social functioning*	*Age and Cancer Stage Adjusted*	0,028	*0*.*630*	*0*.*001*
	Multivariable Adjusted I*	0.020	*0*.*741*	*0*.*031*
	Multivariable Adjusted II**	0.004	*0*.*950*	*0*.*035*
**Symptoms**				
*Fatigue*	*Age and Cancer Stage Adjusted*	-0.080	*0*.*163*	*0*.*006*
	Multivariable Adjusted I*	-0.075	*0*.*217*	*0*.*035*
	Multivariable Adjusted II**	-0.062	*0*.*300*	*0*.*038*
*Nausea and vomiting*	*Age and Cancer Stage Adjusted*	0.019	*0*.*742*	*0*.*000*
	Multivariable Adjusted I*	0.015	*0*.*802*	*0*.*030*
	Multivariable Adjusted II**	0.049	*0*.*407*	*0*.*037*
*Pain*	*Age and Cancer Stage Adjusted*	-0.175	***0*.*002***	*0*.*031*
	Multivariable Adjusted I*	-0.174	***0*.*005***	*0*.*058*
	Multivariable Adjusted II**	-0.131	***0*.*027***	*0*.*051*
*Dyspnea*	*Age and Cancer Stage Adjusted*	-0.115	***0*.*045***	*0*.*013*
	Multivariable Adjusted I*	-0.101	*0*.*098*	*0*.*040*
	Multivariable Adjusted II**	-0.069	*0*.*249*	*0*.*037*
*Insomnia*	*Age and Cancer Stage Adjusted*	-0.114	***0*.*048***	*0*.*013*
	Multivariable Adjusted I*	-0.131	***0*.*029***	*0*.*046*
	Multivariable Adjusted II**	-0.096	*0*.*101*	*0*.*046*
*Appetite loss*	*Age and Cancer Stage Adjusted*	-0.033	*0*.*564*	*0*.*001*
	Multivariable Adjusted I*	-0.034	*0*.*574*	*0*.*032*
	Multivariable Adjusted II**	-0.012	*0*.*836*	*0*.*035*
*Constipation*	*Age and Cancer Stage Adjusted*	0.013	*0*.*827*	*0*.*000*
	Multivariable Adjusted I*	0.009	*0*.*787*	*0*.*023*
	Multivariable Adjusted II**	0.037	*0*.*552*	*0*.*040*
*Diarrhoea*	*Age and Cancer Stage Adjusted*	-0.033	*0*.*568*	*0*.*001*
	Multivariable Adjusted I*	-0.039	*0*.*515*	*0*.*031*
	Multivariable Adjusted II**	-0.021	*0*.*717*	*0*.*036*
*Financial*	*Age and Cancer Stage Adjusted*	-0.036	*0*.*540*	*0*.*001*
	Multivariable Adjusted I*	0.005	*0*.*937*	*0*.*026*
	Multivariable Adjusted II**	-0.021	*0*.*717*	*0*.*036*
*Global Health Status/QoL*	*Age and Cancer Stage Adjusted*	0.010	*0*.*856*	*0*.*020*
	Multivariable Adjusted I*	0.024	*0*.*695*	*0*.*027*
	Multivariable Adjusted II**	-0.032	*0*.*603*	*0*.*036*

*Adjusted for age (continuous), cancer stage (I, II and III), BMI (continuous), type of surgery (quadrantectomy, mastectomy), comorbidities (0, 1, ≥2) and combined therapy (none, at least one);

**Adjusted for age (continuous), cancer stage (I, II and III), smoking status (no, yes, former), step count (continuous), education (continuous), civil status (married or single).

**Table 5:** Association between adherence to the Mediterranean Diet (MedDiet) and HRQoL questionnaires EORTC QLQ-BR23 and EQ-5D-3L.

Questionnaire	Adherence to MedDiet assessed by PREDIMED Questionnaire			
**EQ-5D-3L**				
**EQ-5D-3L score**		*beta*	*p value*	*R*^*2*^
	*Age and Cancer Stage Adjusted*	0.167	***0*.*004***	*0*.*027*
	Multivariable Adjusted I*	0.190	***0*.*003***	*0*.*060*
	Multivariable Adjusted II**	0.113	*0*.*063*	*0*.*047*
**EORTC QLQ C23**				
**Functioning**				
*Body image*	*Age and Cancer Stage Adjusted*	0.076	*0*.*190*	*0*.*008*
	Multivariable Adjusted I*	0.065	*0*.*294*	*0*.*035*
	Multivariable Adjusted II**	0.059	*0*.*329*	*0*.*039*
*Sexual functioning*	*Age and Cancer Stage Adjusted*	-0.037	*0*.*526*	*0*.*001*
	Multivariable Adjusted I*	-0.034	*0*.*584*	*0*.*030*
	Multivariable Adjusted II**	-0.003	*0*.*959*	*0*.*039*
*Future perspective*	*Age and Cancer Stage Adjusted*	-0.058	*0*.*325*	*0*.*003*
	Multivariable Adjusted I*	0.052	*0*.*404*	*0*.*034*
	Multivariable Adjusted II**	0.039	*0*.*524*	*0*.*037*
**Symptoms**				
*Systematic therapy side effects*	*Age and Cancer Stage Adjusted*	-0.080	*0*.*164*	*0*.*006*
	Multivariable Adjusted I*	-0.063	*0*.*293*	*0*.*034*
	Multivariable Adjusted II**	-0.038	*0*.*531*	*0*.*036*
*Breast symptoms*	*Age and Cancer Stage Adjusted*	-0.095	*0*.*086*	*0*.*010*
	Multivariable Adjusted I*	-0.062	*0*.*311*	*0*.*036*
	Multivariable Adjusted II**	-0.054	*0*.*362*	*0*.*039*
*Arm symptoms*	*Age and Cancer Stage Adjusted*	-0.073	*0*.*210*	*0*.*005*
	Multivariable Adjusted I*	-0.063	*0*.*303*	*0*.*035*
	Multivariable Adjusted II**	-0.040	*0*.*500*	*0*.*036*

*Adjusted for age (continuums), cancer stage (I, II and III), BMI (continuous), type of surgery (quadrantectomy, mastectomy), comorbidities (0, 1, ≥2) and combined therapy (none, at least one);

**Adjusted for age (continuous), cancer stage (I, II and III), smoking status (no, yes, former), step count (continuous), education (continuous), civil status (married or single).

In the multivariate analysis adjusted for age and cancer stage we observed that higher adherence to the MedDiet was associated with higher scores for physical functioning (β = 0.199; p = 0.001) and lower scores for pain (β = -0.175; p = 0.002), dyspnea (β = -0.115; p = 0.045) and insomnia (β = -0.114; p = 0.048) in EORTC QLQ-C30 as well as higher scores for well-being from the EQ 5D 3L questionnaire (β = 0.167; p = 0.004). In the multivariate model I additionally adjusted for BMI, type of surgery, combined therapy and comorbidities we observed significant associations with physical functioning (β = 0.207; p = 0.001), pain (β = -0.174; p = 0.005), insomnia (β = -0.131; p = 0.029) and well-being (β = 0.190; p = 0.003). In multivariate model II significant associations were seen for physical functioning (β = 0.169; p = 0.006) and pain (β = -0.131; p = 0.027) after adjustments for age, cancer stage, smoke, step count, education and civil status.

## Discussion

This study found that greater adherence to the MedDiet was associated with higher physical functioning and health status and lower pain and insomnia symptoms suggesting a possible role of the MedDiet in the quality of life of women recently diagnosed with breast cancer. The traditional MedDiet is characterized by high consumption of plant-based foods (vegetables, fruits, whole grains, legumes, nuts, olive oil) and low or limited consumption of red meat, milk and sweets [31].

This dietary pattern has been associated with reduced mortality and lower incidence of chronic diseases including cardiovascular disease, diabetes, cancer, psychological disorders such as depression, reduced pain and improved physical functioning and mental health [1, 2, 32–40]. The MedDiet provides nutrients with demonstrated beneficial health effects such as antioxidants, polyphenols, dietary fiber, polyunsatured and monounsatured fatty acids [41]. Previous studies have investigated possible correlations between the MedDiet, its components and HRQoL in non-oncologic populations. A cross-sectional study in a Spanish population between 2000 and 2005 indicated positive associations between self-perceived mental and physical health and adherence to the MedDiet [42]. Similar results were found in another cross-sectional study from the Moli Sani Project which indicated that higher adherence to a Mediterranean-like eating pattern was related to better HRQoL particularly for mental and physical health [15]. In the SUN Project (Seguimiento Universidad de Navarra) higher adherence to the MedDiet was significantly associated with higher physical functioning and mental health measured after 4 years of follow-up [43]. In the randomized controlled study PREDIMED a direct association was found between baseline adherence to the MedDiet and HRQoL in people with overweight or obesity [16]. Our analysis confirmed previous findings albeit in an oncologic setting. In the HEAL multicenter study of breast cancer survivors the role of lifestyle and nutritional status on HRQoL was investigated and was found that higher diet quality was directly associated with better mental and physical health [44]. These results were confirmed also in other studies with cancer patients [45]. In the Iowa Women's Health Study (IWHS) of elderly cancer survivors, better quality of life was related with higher adherence to the WCRF/AICR recommendations which included typical aspects of the MedDiet (i.e. higher consumption of vegetables and fruit and lower consumption of fast-foods, red meat, and sugar-sweetened drinks) [46, 47]. A generally healthy dietary pattern which included vegetables, whole grains, fruit, fish, yogurt and soy was also associated with better scores for dyspnea but worse scores for insomnia [48]. Unlike these authors we found better scores for both dyspnea and insomnia with higher adherence to MedDiet. Lack of sleep or poor quality of sleep may alter physical and mental functioning may lead to increased pain or pain perception in cancer patients [49–52]. A possible mechanism of the relationship between a healthy diet and quality of life may be through inflammation [53, 54] and the MedDiet has been previously associated with reduced inflammation [55].

Large consumption of plant-based foods, olive oil, fruits and nuts provides antioxidants, polyphenols and monounsaturated fatty-acids which may positively impact on inflammation, endothelial function and pain [56–59]. A higher well-being is of paramount importance since it may lead to better adherence to oncologic treatment and to higher physical activity which are known to reduce breast cancer recurrence and mortality [8, 60, 61].

This study has some limitations. The cross-sectional nature of this baseline analysis does not allow to infer cause-effect but only possible associations nor to rule out reverse associations. When people feel well they may eat better, while feeling unwell may lead to consume more comfort foods (e.g. sweets). The sample size is relatively small therefore the study cannot have the power and generalizability of a large-scale epidemiologic investigation in breast cancer patients. Patients were enrolled at different stages of cancer treatment which may have differently affected HRQoL however we adjusted for cancer therapy. Although response-shift may affect quality of life outcomes [62], our study included patients enrolled within one year of surgery and time from surgery did not show any effect modification. We were also able to account for type of breast cancer surgery and for comorbidities which may both affect quality of life. Finally, our study is the first to analyse and report in details the relationship between all components of the HRQoL questionnaires and adherence to the MedDiet utilizing a multivariate adjusted analysis.

This study suggests that higher adherence to the MedDiet may impact positively on quality of life of breast cancer survivors living in a Mediterranean country, specifically for physical functioning, sleep, pain and overall well-being, confirming results found in healthy individuals.

These findings are relevant in breast cancer survivors whose lower quality of life may negatively affect treatment compliance and disease outcomes.

## Supporting information

S1 Dataset (XLS)
