# Correction: Mediterranean diet and quality of life in women treated for breast cancer: A baseline analysis of DEDiCa multicentre trial

**DOI:** 10.1371/journal.pone.0256944

**Published:** 2021-08-26

**Authors:** Giuseppe Porciello, Concetta Montagnese, Anna Crispo, Maria Grimaldi, Massimo Libra, Sara Vitale, Elvira Palumbo, Rosa Pica, Ilaria Calabrese, Serena Cubisino, Luca Falzone, Luigina Poletto, Valentina Martinuzzo, Melania Prete, Nadia Esindi, Guglielmo Thomas, Daniela Cianniello, Monica Pinto, Michelino De Laurentiis, Carmen Pacilio, Massimo Rinaldo, Massimiliano D’Aiuto, Diego Serraino, Samuele Massarut, Chiara Evangelista, Agostino Steffan, Francesca Catalano, Giuseppe L. Banna, Giuseppa Scandurra, Francesco Ferraù, Rosalba Rossello, Giovanna Antonelli, Gennaro Guerra, Amalia Farina, Francesco Messina, Gabriele Riccardi, Davide Gatti, David J. A. Jenkins, Anita Minopoli, Bruna Grilli, Ernesta Cavalcanti, Egidio Celentano, Gerardo Botti, Maurizio Montella †, Livia S. A. Augustin

The affiliation for the twenty-fifth author is incorrect. Chiara Evangelista is not affiliated with #4 but with #1: Immunopathology and Cancer Biomarkers Unit, National Cancer Institute Centro di Riferimento Oncologico IRCCS, Aviano, Italy.
